# GPR87 Promotes Metastasis through the AKT-eNOS-NO Axis in Lung Adenocarcinoma

**DOI:** 10.3390/cancers14010019

**Published:** 2021-12-21

**Authors:** Hye-Mi Ahn, Eun-Young Choi, Youn-Jae Kim

**Affiliations:** Targeted Therapy Branch, Division of Rare and Refractory Cancer, Research Institute, National Cancer Center, Goyang 10408, Gyeonggi, Korea; 75974@ncc.re.kr (H.-M.A.); eyc@ncc.re.kr (E.-Y.C.)

**Keywords:** lung adenocarcinoma, GPR87, GPCR, eNOS, NO, AKT

## Abstract

**Simple Summary:**

Lung adenocarcinoma is one of the leading causes of cancer-related deaths. Even though advanced anticancer drugs are available, prognosis of patients with lung cancer is dismal and there is an urgent need to explore novel oncogenic mechanisms to overcome these therapeutic limitations. GPR87 is upregulated in various cancers, but its biological function has not yet been established in lung cancer. Here, we discovered that GPR87 is upregulated in lung adenocarcinoma and overexpressed GPR87 contributes to poor prognosis in patients with lung adenocarcinoma. We used GPR87-overexpressing and GPR87-silenced lung adonocarcinoma cell lines, along with in vivo studies, to demonstrate that overexpression of GPR87 promoted invasiveness and metastasis of lung adenocarcinoma cells. Our study identified the AKT-eNOS-NO signaling axis to be the mechanism by which GPR87 exerted its oncogenic function.

**Abstract:**

Lung adenocarcinoma is one of the leading causes of cancer-related deaths. Despite the availability of advanced anticancer drugs for lung cancer treatment, the prognosis of patients still remains poor. There is a need to explore novel oncogenic mechanisms to overcome these therapeutic limitations. The functional experiments in vitro and in vivo were performed to evaluate the role of GPR87 expression on lung adenocarcinoma metastasis. The public lung adenocarcinoma dataset was used to determine the clinical relevance of GPR87 expression in patients with lung adenocarcinoma. GPR87 is upregulated in various cancer; however, the biological function of GPR87 has not yet been established in lung adenocarcinoma. In this study, we found that GPR87 expression is upregulated in lung adenocarcinoma and is associated with poor patient prognosis. Additionally, we showed that GPR87 overexpression promotes invasiveness and metastasis of lung adenocarcinoma cells. Furthermore, we demonstrated that AKT-eNOS-NO signaling is a novel downstream pathway of GPR87 in lung adenocarcinoma. Conversely, we confirmed that silencing of GPR87 expression suppressed these phenotypes. Our results reveal the oncogenic function of GPR87 in cancer progression and metastasis through the activation of eNOS as a key mediator. Therefore, we propose that targeting eNOS could be a novel therapeutic strategy to improve the clinical treatment of lung adenocarcinoma.

## 1. Introduction

Lung cancers are typically categorized into two main types: non-small cell lung cancer (NSCLC) and small cell lung cancer (SCLC). NSCLC accounts for more than 85% of lung cancers and is further subclassified into lung adenocarcinoma, squamous cell carcinoma, and large cell carcinoma [1]. Among them, lung adenocarcinoma accounts for approximately ~80% of NSCLC cases [1]. According to GLOBOCAN 2020 data analyzing cancer incidence and mortality reported by the International Agency for Research on Cancer, lung adenocarcinoma is currently the most frequent and leading cause of cancer-related deaths worldwide [1,2]. Despite the development of advanced anticancer drugs to treat lung cancer, the prognosis of patients with lung cancer still remains poor [3,4]. Metastasis of the primary tumor to distant organs and resistance to therapeutic agents may be among several reasons for the poor prognosis associated with this subtype [5,6]. Therefore, the identification of appropriate targets and elucidation of the oncogenic mechanisms underlying metastasis are critical for the development of novel therapeutic strategies for lung adenocarcinoma.

G protein-coupled receptors (GPCRs) are a large family of proteins containing seven transmembrane domains and are encoded by of over 800 genes [7,8]. Since many GPCRs are known to be involved in the pathological mechanisms of various diseases, they are important targets for the development of therapeutic agents [9,10]. Aberrant overexpression of GPCRs has been reported in various tumor tissues and it promotes cancer progression and drug resistance [11]. G protein-coupled receptor 87 (GPR87) is a member of the GPCR family and has also been shown to be overexpressed in many tumor tissues, including breast, bladder, pancreatic, and liver cancer. GPR87 overexpression promotes cancer cell proliferation [12,13], survival [14,15], tumor development [16,17], and metastasis [18]. In pancreatic cancer, GPR87 enhances aggressiveness by activating the NF-κB signaling pathway [17] and stem cell expansion via activation of the JAK2/STAT3 signaling pathway [19]. However, since the functional role of GPR87 expression has not been established in lung adenocarcinoma, the regulatory mechanisms of GPR87 in lung adenocarcinoma development and progression remain to be elucidated.

In the present study, we identified that GPR87 is an important effector in promoting malignancy in lung adenocarcinoma. We found that GPR87 was upregulated in lung adenocarcinoma tissues and was significantly associated with poor prognosis of patients with lung adenocarcinoma. Overexpression of GPR87 promoted cancer cell invasion and a metastatic phenotype, while depletion of GPR87 suppressed these effects. Our results elucidate the mechanism for the oncogenic function of GPR87 in the progression and metastasis of lung adenocarcinoma, and suggest strategies that may improve therapeutic outcomes.

## 2. Materials and Methods

### 2.1. Cell Culture and Transfection

A549 Red-FLuc lung adenocarcinoma cells were purchased from PerkinElmer (Waltham, MA, USA) and A427 lung adenocarcinoma cells were purchased from the Korean Cell Line Bank (KCLB, Seoul, Korea). Cells were maintained in RPMI-1640 (Cytiva, Marlborough, MA, USA) medium supplemented with 1% penicillin-streptomycin (Welgene, Gyeongsan, Korea), 1% sodium pyruvate and 10% fetal bovine serum (FBS, Cytiva, Marlborough, MA, USA) at 37 °C in a humidified atmosphere of 5% CO2.

For transfection of the GPR87 overexpression or control vectors, 1.5 ~ 3 × 10^5^ lung cancer cells were seeded into six-well plates and incubated overnight. Control empty or GPR87 expression vector at a concentration of 1 µg were transfected into lung cancer cells using Lipofectamine 2000 in Opti-MEM medium. After 4 h of incubation, the medium was changed to complete medium, and 48 h following transfection, overexpression was confirmed by western blotting. siRNA target sequences were designed using the AsiDesigner program. For siRNA transfection, 1.5 × 10^5^ lung cancer cells were seeded into six-well plates and incubated overnight. siRNAs and non-targeting controls at a concentration of 100 nM were transfected into lung cancer cells using Lipofectamin 2000 in Opti-MEM medium. After 4 h of incubation, the medium was changed to complete medium, and 48 h following transfection, knockdown was confirmed by qRT-PCR. 

### 2.2. Invasion Assay

Transwell chambers (Corning, New York, NY, USA) were coated with Matrigel Basement Membrane Matrix (BD Biosciences, East Rutherford, NJ, USA). Cells were suspended in serum-free medium and seeded into the upper chamber at a density of 2.0 to 5.0 × 10^4^ cells per well, and serum-containing medium were placed into the lower chamber. Following an incubation of 24 h, cells penetrating the pores were stained with Diff-Quick staining solution (Sysmex, Kobe, Japan) and observed using a microscope.

### 2.3. Proliferation Assay

Cells were seeded in 96-well plates at a density of 2 × 10^3^ cells/well. After 0–72 h, a mixture of CyQUANT NF Cell Proliferation dye reagent and deliverer (Invitrogen, Waltham, MA, USA) was added and incubated at 37 °C for 30 min. Fluorescence intensity was measured as the ratio of the fluorescence at 530 nm to that at 485 nm using an infinite M200 Pro microplate reader (TECAN, Männedorf, Switzerland).

### 2.4. Western Blot Analysis

Whole-cell lysates were prepared using RIPA buffer (iNtRON Biotechnology, Seongnam, Korea) with a protease inhibitor cocktail (Roche, Basel, Switzerland). Total protein samples were quantified using the Pierce BCA Protein Assay Kit (Thermo, Waltham, MA, USA). Equal amounts of protein lysates were separated on 8–16% Bis-Tris protein gels (Invitrogen, Waltham, MA, USA), transferred to PVDF membranes (Millipore, Burlington, MA, USA), and blocked with 5% skim milk (BD Biosciences, NJ, USA). The following primary antibodies were used: anti-GPR87 (Abcam, Cambridge, UK), anti-N-cadherin, anti-ZEB1, anti-E-cadherin, anti-Claudin, anti-phospho-AKT, anti-AKT, anti-phospho-eNOS, anti-eNOS and horseradish peroxidase (HRP)-conjugated anti-β-actin, (Cell Signaling, Danvers, MA, USA). An HRP-conjugated anti-mouse IgG antibody (Bio-Rad, Hercules, CA, USA) was used as the secondary antibody. Specific bands were detected using the WEST-ZOL plus Western Blot Detection System (iNtRON Biotechnology, Seongnam, Korea).

### 2.5. Phospho-Kinase Array

Cells were seeded in 100 mm plates at a density of 2 × 10^6^ cells and incubated overnight. The medium was changed to serum free medium for overnight and treated with lysophosphatidic acid (LPA) for 15 min. Following treatment, the cells were washed with PBS and lysates were prepared using a lysis buffer. Equal amounts of about 500 μg of protein lysates were applied each array membrane. The treated cells were performed using a Human Phospho-Kinase Array Kit (R&D Systems, Minneapolis, MI, USA) to detect the phospho-kinase activity according to the manufacturer’s protocol.

### 2.6. Nitrite Assay

Cells were seeded in 60 mm plates at a density of 2 × 10^6^ cells and incubated overnight. The medium was changed to serum free medium for overnight and treated with LPA for 24 h. The concentrated supernatant was performed using a Nitrite/Nitrate colorimetric assay kit (SIGMA, Ronkonkoma, NY, USA) to detect the intracellular nitric oxide (NO) production according to the manufacturer’s protocol.

### 2.7. In Vivo Tumor Models

This study was reviewed and approved by the Institutional Animal Care and Use Committee (IACUC) of National Cancer Center Research Institute. NCCRI is an Association for Assessment and Accreditation of Laboratory Animal Care International (AAALAC International) accredited facility and abide by the Institute of Laboratory Resources (ILAR) guide. Nude mice (BALB/c-nude, five weeks old females) were purchased from OrientBio (Seongnam, Korea). After a week, 1.0 × 10^6^ lung cancer cells resuspended in 100 μL phosphate-buffered saline (PBS) were intravenously administered via tail vein injection. Tumor metastasis was monitored by weekly bioluminescence imaging using the IVIS Lumina III (PerkinElmer, USA). Mice were sacrificed 27 days following cancer cell injection.

### 2.8. Evaluation of Lung Metastasis

Lung metastasis of the transplanted tumor was observed under a microscope. The number of metastatic nodules of the transplanted tumor in the lung was counted. Some lung tissues were prepared for H&E staining to determine the lung metastasis of the transplanted tumor.

### 2.9. Public Data Analysis

Public microarray data containing clinical information were downloaded from the Oncomine database (http://www.oncomine.org, accessed on 3 April 2021). The accession numbers of collected data were GSE19188 and GSE10072. For the survival analyses, two datasets (GSE50081 and GSE30219) were downloaded from the Kaplan-Meier Plotter (https://kmplot.com/analysis/, accessed on 3 April 2021) site.

### 2.10. Statistical Analysis

Data were presented as the mean ± standard deviation (SD) from three independent experiments. Statistical analyses were performed with a Student’s t-test and the log-rank test. * *p* < 0.05; ** *p* < 0.01; *** *p* < 0.001 were considered statistically significant.

## 3. Results

### 3.1. GPR87 Promotes Invasiveness of Lung Adenocarcinoma Cells

**Figure 1:**
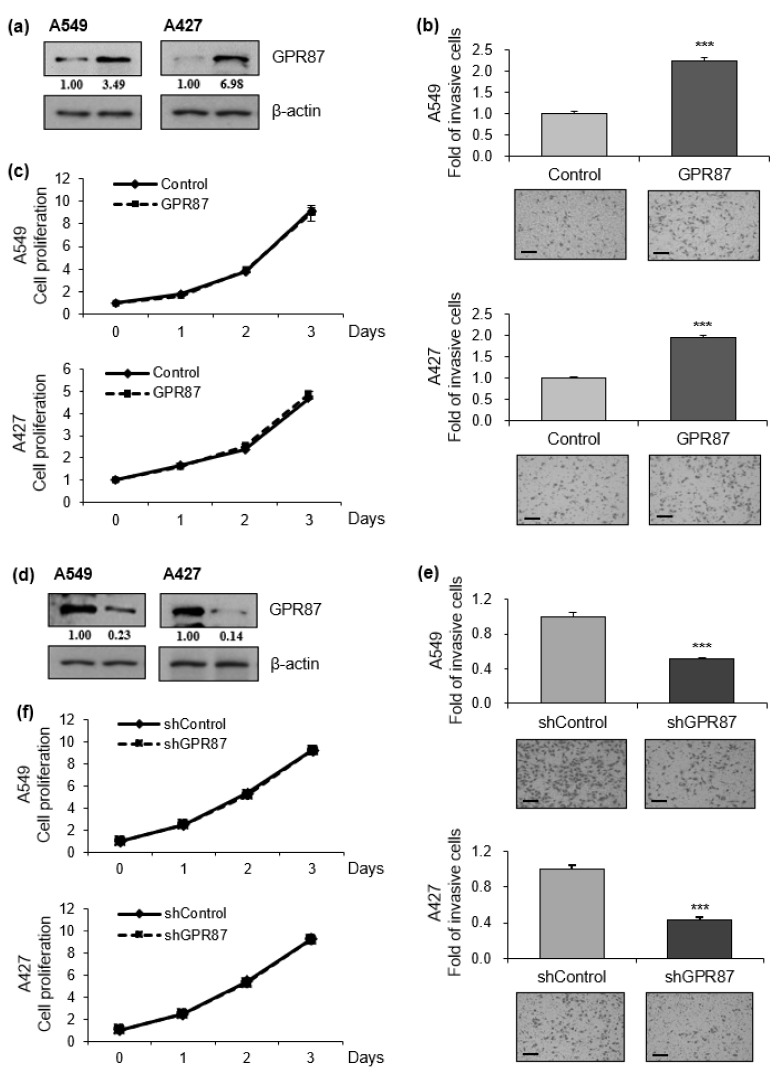
GPR87 promotes lung adenocarcinoma cell invasion. (**a**) Western blot of A549 and A427 cells transfected with empty control or GPR87-overexpressing vector. (**b**) Invasion assays of A549 and A427 cells transfected with empty control or GPR87-overexpressing vector (**c**) Cell viability assays of A549 and A427 cells transfected with empty control or GPR87-overexpressing vector. (**d**) Western blot of A549 and A427 cells transfected with shcontrol or shGPR87. (**e**) Invasion assays of A549 and A427 cells transfected with shcontrol or shGPR87. (**f**) Cell viability assays of A549 and A427 cells transfected with shcontrol or shGPR87. The number of invading cells in each field of view were counted and quantified. Values represent mean ± standard deviation (SD) from three independent experiments. *** *p* < 0.001 vs control. Scale bar = 200 µm.

To evaluate the functional role of GPR87 overexpression in lung adenocarcinoma progression, we performed invasion and proliferation assays in GPR87-overexpressing lung adenocarcinoma cell lines (Figure 1a and Figure S1a,b). We found that GPR87-overexpressing A549 and A427 cells displayed dramatically increased cancer cell invasion compared to control cells (Figure 1b). However, GPR87 overexpression did not affect the proliferation of lung adenocarcinoma cells (Figure 1c). To further determine whether downregulation of GPR87 inhibited cell proliferation and invasion in lung adenocarcinoma, we performed invasion and proliferation assays in GPR87-silenced lung adenocarcinoma cells (Figure 1d and Figure S1c,d). Downregulation of GPR87 decreased cancer cell invasion but did not affect cell proliferation in A549 and A427 cells (Figure 1e,f). Based on these results, we inferred that GPR87 overexpression promotes lung adenocarcinoma cell invasiveness and plays a critical oncogenic role in lung adenocarcinoma progression.

### 3.2. GPR87 Regulates Mesenchymal Phenotype in Lung Adenocarcinoma Cells

**Figure 2:**
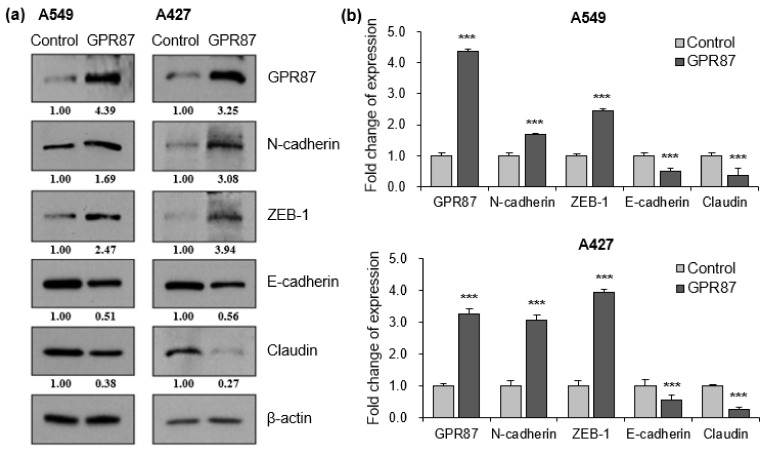

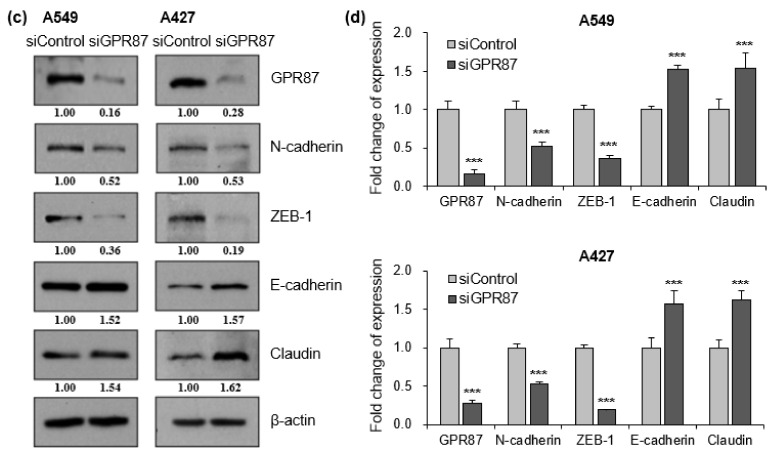
GPR87 regulates expression of EMT markers and EMT-inducing transcription factor. (**a**) Western blot of N-cadherin, ZEB-1, E-cadherin, and claudin expression in A549 and A427 cells transfected with empty control or GPR87-overexpressing vector. (**b**) Relative expression of protein levels shown in (a), normalized to β-actin. (**c**) Western blot of N-cadherin, ZEB-1, E-cadherin, and claudin expression in A549 and A427 cells transfected with sicontrol or siGPR87. (**d**) Relative expression of protein levels shown in (**c**), normalized to β-actin. Values represent mean ± standard deviation (SD) from three independent experiments. *** *p* < 0.001 vs. control.

The epithelial to mesenchymal transition (EMT) is a critical process in the initiation of tumor development. During tumor progression, cell-cell adhesion and tight junctions decreases, while cell motility increases [20,21]. Since EMT contributes to the poor prognosis of cancer patients, we examined whether the expression of GPR87 affects EMT in lung adenocarcinoma cells. We performed western blot analysis to determine the expression of EMT markers, wherein the EMT-inducing transcription factor was regulated by GPR87 overexpression. GPR87 overexpression markedly increased the expression of the mesenchymal cell marker, N-cadherin and the EMT-inducing transcription factor, ZEB-1 (Figure 2a,b and Figure S2a). Conversely, GPR87 overexpression markedly reduced the expression of the epithelial cell markers E-cadherin and claudin (Figure 2a,b and Figure S2a). Next, we evaluated the impact of GPR87 depletion on the expression of EMT markers and EMT-inducing transcription factors. As expected, GPR87 suppression markedly increased the expression of the epithelial cell markers E-cadherin and claudin (Figure 2c,d and Figure S2b). On the contrary, GPR87 suppression markedly reduced the expression of the mesenchymal cell marker, N-cadherin and EMT-inducing transcription factor, ZEB-1 (Figure 2c,d and Figure S2b). These results suggest that GPR87 regulates EMT and expression of EMT-related genes.

### 3.3. GPR87 Promotes Malignant Features by Activating AKT-eNOS Signaling

**Figure 3:**
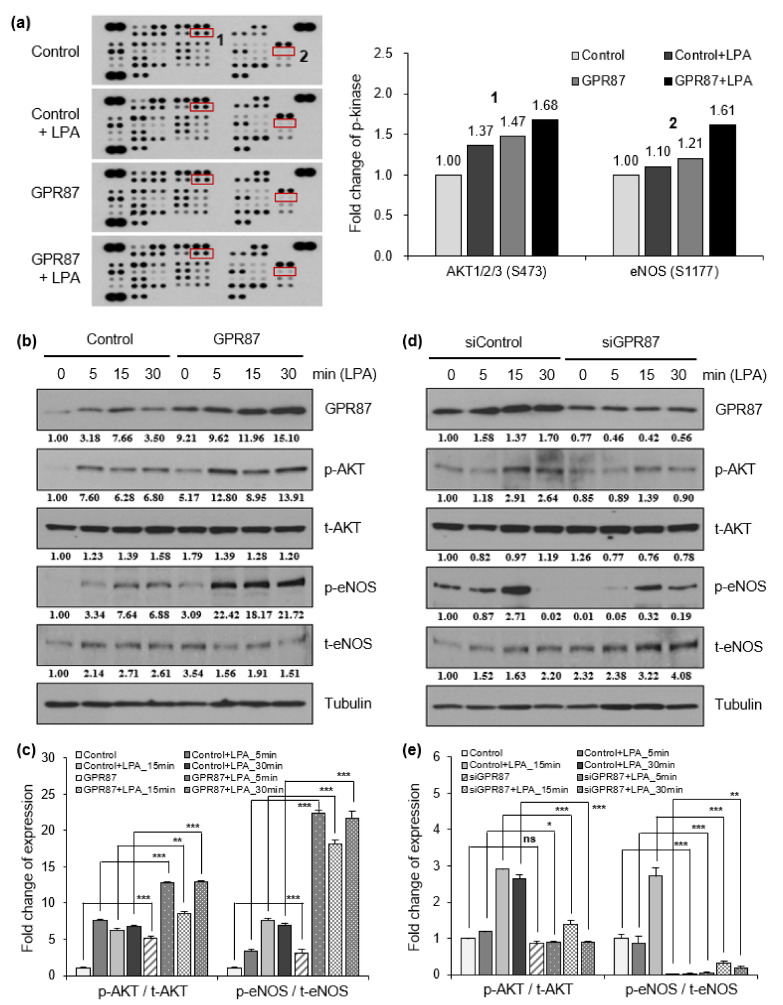
GPR87 activates AKT-eNOS signaling, and induces NO production. (**a**) Results of human phospho-kinase array in A549 cells transfected with empty control or GPR87-overexpressing vector and treated with LPA (10 µM). (**b**) Western blot of GPR87, phosphorylated AKT and eNOS, and total levels of AKT and eNOS were determined in A549 cells transfected with empty control or GPR87-overexpressing vector and treated with LPA (10 µM). Tubulin was used as the loading control. (**c**) Relative eNOS and AKT phosphorylation levels normalized to eNOS and AKT total protein levels shown (**b**). (**d**) Western blot of GPR87, phosphorylated AKT and eNOS, and total levels of AKT and eNOS were determined in transfected with sicontrol and GPR87-silenced A549 cells treated with LPA (10 µM). Tubulin was used as the loading control. (**e**) Relative eNOS and AKT phosphorylation levels normalized to eNOS and AKT total protein levels shown in (**d**). Values represent mean ± standard deviation (SD) from three independent experiments. * *p* < 0.05, ** *p* < 0.01, *** *p* < 0.001 vs. control.

To investigate the mechanism by which GPR87 promotes lung adenocarcinoma cell progression and metastatic ability, a human phospho-kinase array was performed using A549 lung adenocarcinoma cells. Since the results from the two cell lines were very similar, we used A549 lung adenocarcinoma cells in subsequent experiments. GPCRs are known to be involved in various intracellular processes such as cell growth, proliferation, survival, and tumorigenesis through the AKT signaling pathway [22]. A potential ligand of GPR87, LPA, induced AKT phosphorylation (Figure 3a and Figure S3a). GPR87 overexpression further increased the effects of LPA treatment. Interestingly, we found that phosphorylation of eNOS was activated by overexpression of GPR87 and LPA treatment (Figure 3a and Figure S3a). Western blot analysis confirmed that LPA treatment significantly increased AKT and endothelial nitric oxide (eNOS) phosphorylation, and GPR87 overexpression further increased the effect of LPA treatment in A549 lung cancer cells (Figure 3b,c and Figure S3b). We further investigated whether suppression of GPR87 inhibited AKT-eNOS signaling. After confirming the downregulation of GPR87 in GPR87-silenced cells, we examined the effect of GPR87 silencing on downstream signaling in A549 lung adenocarcinoma cells. LPA promoted AKT and eNOS phosphorylation, and silencing of GPR87 significantly reduced the increased phosphorylation of AKT and eNOS induced by LPA treatment (Figure 3d,e and Figure S3c). Taken together, these results indicate that GPR87 promotes malignant properties by activating AKT-eNOS signaling in lung adenocarcinoma cells.

### 3.4. eNOS Is a Key Mediator for GPR87 in Regulating Metastatic Properties in Lung Adenocarcinoma Cells

**Figure 4:**
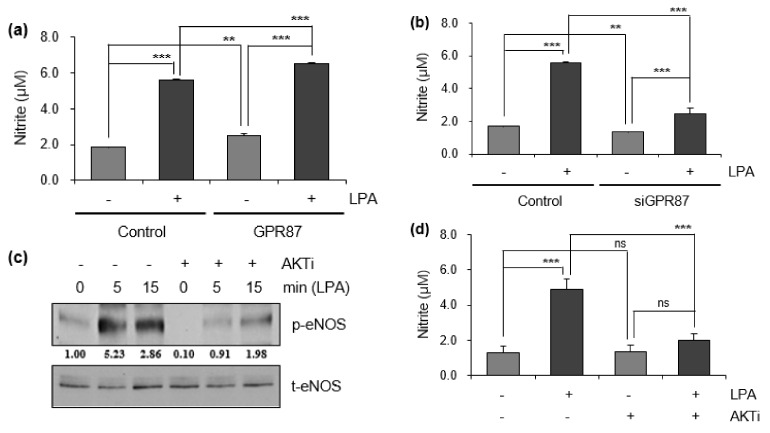

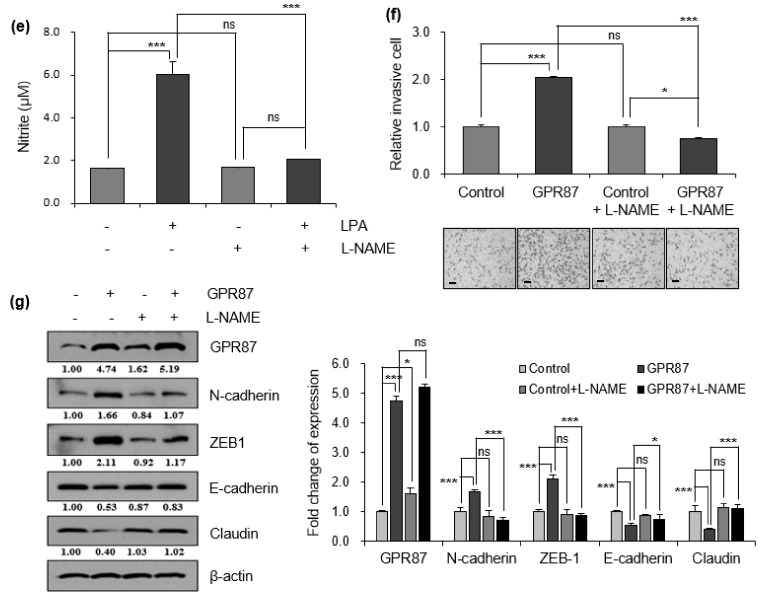
GPR87 promotes lung adenocarcinoma cell invasion through the activation of AKT-eNOS signaling. (**a**) The level of intracellular NO was determined in GPR87-overexpressing A549 cells treated with LPA (10 µM). (**b**) The level of intracellular NO was determined in GPR87-silenced A549 cells treated with LPA (10 µM). (**c**) The level of phosphorylated eNOS was determined in A549 and PF-04691502 (1 µM) pre-treated A549 cells after treatment with LPA (10 µM). (**d**) In A549 and PF-04691502 (1 µM) pre-treated A549 cells, the level of intracellular NO was determined after treatment with LPA (10 µM). (**e**) In A549 and L-NAME (1 mM) pre-treated A549 cells, the level of intracellular NO was determined after treatment with LPA (10 µM). (**f**) In A549 and GPR87-overexpressing A549 cells, invasion assay was performed after treatment with L-NAME (1 mM). The number of invaded cells were counted for each field and quantified. (**g**) Expression levels of N-cadherin, ZEB-1, E-cadherin, and claudin in A549 and GPR87-overexpressing A549 cells after treatment with L-NAME (1 mM). Values represent mean ± standard deviation (SD) from three independent experiments. * *p* < 0.05, ** *p* < 0.01, *** *p* < 0.001 vs control. Scale bar = 200 µm.

Next, we determined whether LPA treatment and GPR87 overexpression had an effect on NO production in A549 lung adenocarcinoma cells. As shown in Figure 4a, intracellular NO production was induced by LPA treatment and GPR87 overexpression in A549 lung adenocarcinoma cells. Moreover, the level of intracellular NO in GPR87-overexpressing A549 cells further increased the effect of LPA treatment. We also investigated whether suppression of GPR87 inhibited NO production. We confirmed the downregulation of GPR87 in GPR87-silenced A549 cells. Compared to the LPA-treated cells, downregulation of GPR87 significantly abolished the LPA-induced intracellular NO production (Figure 4b). The AKT kinase inhibitor, PF-04691502 was used to examine the role of AKT signaling in regulating NO production in A549 lung adenocarcinoma cells. Compared to the LPA-treated cells, treatment with PF-04691502 significantly diminished LPA-induced upregulation of eNOS phosphorylation (Figure 4c and Figure S4c). Additionally, treatment with PF-04691502 significantly reduced intracellular NO production in A549 cells (Figure 4d). These results showed that GPR87 overexpression induced the phosphorylation of AKT and eNOS, and increased intracellular NO production. Intracellular NO is closely associated with tumorigenesis and cancer progression. Furthermore, to confirm whether GPR87 promotes metastatic abilities through eNOS activation in A549 lung adenocarcinoma cells, L-NAME, an inhibitor of eNOS, was used. Compared to the LPA-treated cells, treatment with L-NAME notably reduced the LPA-induced intracellular NO production (Figure 4e). Consistently, L-NAME treatment significantly inhibited A549 lung adenocarcinoma cell invasion by GPR87 overexpression (Figure 4f). We also evaluated the impact of blockade of eNOS activity on the expression of EMT markers and EMT-inducing transcription factors in A549 lung adenocarcinoma cells. As expected, L-NAME treatment markedly abolished the effects of GPR87 overexpression (Figure 4g and Figure S4g). Collectively, our results indicate that eNOS is a key mediator for GPR87 in the regulation of metastatic properties through the AKT-NO signaling pathway in lung adenocarcinoma. 

### 3.5. GPR87 Promotes Lung Adenocarcinoma Metastasis and Is Correlated with Poor Prognosis

**Figure 5:**
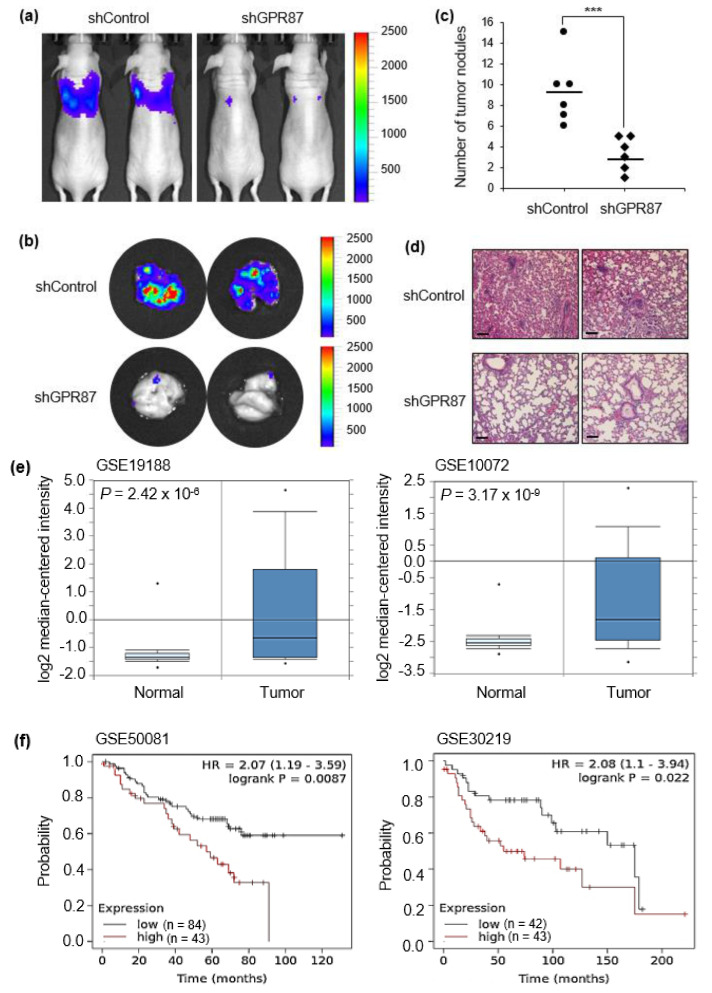
GPR87 is correlated with cancer progression and poor prognosis of lung adenocarcinoma. (**a**) Visualization of the luminescence signal of GPR87-silenced A549-derived metastatic cells in the lungs of mice following intravenous injection. (**b**) Visualization of the luminescence signal of GPR87-silenced A549-derived metastatic cells in the lungs of mice after sacrifice. (**c**) The number of metastatic nodules was determined in the lungs. (**d**) H&E staining of metastatic nodules in the lungs. (e) Analysis of GPR87 mRNA expression in normal lung and lung adenocarcinoma tissues from the Oncomine database (*p* = 2.42 × 10^−6^ for GSE19188 and *p* = 3.17 × 10^−9^ for GSE10072, *t*-test). (**f**) The overall survival rates of lung adenocarcinoma patients classified by GPR87 expression (*p* = 8.7 × 10^−3^, *n* = 127 for GSE50081 and *p* = 2.2 × 10^−2^, *n* = 85 for GSE30219, log-rank test). Values represent mean ± standard deviation (SD) from three independent experiments. *** *p* < 0.001 vs control. Scale bar = 200 µm.

To evaluate the clinical effect of GPR87 silencing on lung metastasis, we used an in vivo tumor model. GPR87-silenced A549-FLuc lung adenocarcinoma cells were intravenously injected into the tail veins of nude mice, and the luminescence signal was visualized once a week. As shown in Figure 5a,b, mice injected with GPR87-silenced cells exhibited significantly suppressed adenocarcinoma cell metastasis in the lung. Downregulation of GPR87 decreased the number of metastatic nodules in mice lungs (Figure 5c). In addition, hematoxylin and eosin (H&E) staining analysis was performed in lung tissues (Figure 5d). These results indicate that GPR87 depletion suppresses the metastatic properties of lung adenocarcinoma cells. To investigate the clinical relevance of GPR87 expression in lung adenocarcinoma patients, we analyzed public microarray lung adenocarcinoma datasets from the NCBI Gene Expression Omnibus (GEO) database. Two datasets for lung adenocarcinomas, GSE19188 and GSE10072, were used in this study. We compared the expression of GPR87 in normal and tumor lung tissues and found that the expression was significantly upregulated in the tumor tissues in both of the datasets (*p* = 2.42 × 10^−6^ for GSE19188 and *p* = 3.17 × 10^−9^ for GSE10072, *t*-test, Figure 5e). Using the Kaplan-Meier survival analysis, we examined the association between GPR87 expression and survival outcomes of patients with lung adenocarcinoma. Overall survival was remarkably shorter in patients with high GPR87 expression than in those with low GPR87 expression (*p* = 8.7 × 10^−3^ for GSE50081 and *p* = 2.2 × 10^−2^ for GSE30219, *t*-test, Figure 5f). These findings suggest that GPR87 overexpression is associated with cancer progression and poor prognosis in patients with lung adenocarcinoma.

## 4. Discussion

In the present study, our findings provide evidence that GPR87 overexpression significantly promotes invasive ability in lung adenocarcinoma cells. Conversely, we reveal that silencing of GPR87 markedly inhibits invasive ability. These results suggest that GPR87 plays an oncogenic role in lung cancer progression. Previous studies have reported that GPR87 overexpression promotes invasiveness of liver [18] and pancreatic cancer cells [17], whereas loss of GPR87 inhibits invasive ability in pancreatic cancer cells [17]. Our results are consistent with these findings and support the tumor-promoting effect of GPR87 in lung adenocarcinoma. Moreover, we show that the expression of EMT markers was regulated according to the expression of GPR87. These results suggest that GPR87 overexpression promotes EMT and may eventually alter the invasiveness of lung adenocarcinomas. In this study, the association between GPR87 expression and EMT-related genes was demonstrated for the first time in lung adenocarcinoma. Furthermore, to investigate the clinical relevance of GPR87 in lung adenocarcinoma, we analyzed public microarray datasets. Our data indicate that GPR87 overexpression is associated with cancer progression and poor survival outcomes.

GPR87 was first reported by Nonkada et al. [23], and it is known to be a cognate receptor activated by LPA [24,25]. GPR87 is upregulated in various tumors and plays an important role in cancer cell survival [14]. GPR87 overexpression promotes cancer cell survival by activating AKT and repressing p53, thereby inhibiting apoptotic potential in bladder cancer [15,26]. Conversely, inhibition of GPR87 in lung cancer suppresses tumor proliferation by reducing the expression of KRAS and c-MYC [12,13]. Furthermore, GPR87 overexpression promotes cancer cell growth and metastasis by upregulating CD133-positive cancer stem-like cells in liver cancer [18]. However, the biological mechanism of GPR87 in lung adenocarcinoma has not been clearly elucidated. We therefore, investigated the downstream signaling pathway through which GPR87 promotes lung adenocarcinoma progression by performing a human phospho-kinase screening. We found that LPA treatment significantly increased AKT and eNOS phosphorylation and GPR87 overexpression further increased the effect of LPA treatment. Furthermore, suppression of GPR87 inhibited the effect of LPA treatment. Our data imply that GPR87 promotes malignant characteristics by activating AKT-eNOS signaling in lung adenocarcinoma cells. In general, the functional role of eNOS in cancer biology is not yet understood. In the present study, we elucidated the mechanism of the GPR87-AKT-eNOS-NO pathway for the first time in lung adenocarcinoma; however, further studies are needed to determine whether the GPR87 pathway is the same in other types of cancer.

Based on our results, we speculate that GPR87 enhances NO production by stimulating eNOS activation, thereby promoting the progression of lung adenocarcinoma. Nitric oxide synthases (NOSs) consist of three subtypes: eNOS (endothelial nitric oxide synthase), iNOS (inducible nitric oxide synthase), and nNOS (neuronal nitric oxide synthase) [27]. NO is a highly reactive gaseous product and is produced by the conversion of L-arginine to L-citrulline by NOSs [28,29]. As a signal transduction molecule, NO plays a critical role in diverse physiological and pathological processes, including cancer biology [30,31]. Studies have reported that NO is associated with angiogenesis, apoptosis, invasiveness, and metastasis in many carcinomas [32,33,34,35]. Many studies conducted so far in lung adenocarcinoma have mostly examined the biological function of iNOS, and few studies have been conducted on eNOS.

It is well established that eNOS activity is regulated by post-translational modifications, such as serine/threonine kinase AKT phosphorylation and intracellular calcium levels [36,37,38]. Under normal conditions, NO synthesized by eNOS in endothelial cells regulate anti-oxidative and anti-inflammatory responses [27,39]. Recently, several studies have been conducted to investigate the functional role of eNOS expressed in cancer. Oncogenic KRas promotes pancreatic cancer cell growth through the activation of PI3K-AKT-eNOS signaling [40]. In addition, eNOS-generated NO regulates angiogenesis in a xenograft model and the inhibition of eNOS may be a worthy therapeutic target in pancreatic cancer [41]. In cholangiocarcinoma, the activation of eNOS significantly enhances migration and invasion via the phosphorylation of vasodilator-stimulated protein (VASP) [42]. Our results are consistent with these findings and we have demonstrated that eNOS is a key mediator for GPR87 in the regulation of metastatic properties through the AKT-eNOS-NO signaling pathway in lung adenocarcinoma. These findings revealed that eNOS may play a critical role in cancer cells, not endothelial cells, and further emphasize the importance of understanding the underlying role of eNOS.

To evaluate the clinical effect of GPR87 on lung metastasis, we used a xenograft mouse model. When GPR87-silenced lung adenocarcinoma cells were intravenously injected into the tail veins of nude mice, our results indicated that GPR87 depletion suppressed the metastasis of lung adenocarcinoma cells. One limitation of our study may be that the tail vein injection model is not an accurate in vivo model for the study of lung cancer metastasis. Well- defined preclinical models are needed to better elucidate the biological function of GPR87 in metastasis of lung adenocarcinoma [43,44]. According to a recently published paper, humanized anti-GPR87 mAb had anti-tumor effects in a mouse model of lung cancer [45]. This study supports our results, highlighting the oncogenic function of GPR87 in lung adenocarcinoma. In the present study, we elucidated the functional role and molecular mechanism of GPR87 in lung adenocarcinoma. We also found that L-NAME treatment markedly abolished the effects of GPR87 overexpression, indicating that the inhibition of eNOS can inhibit GPR87-induced metastatic properties in lung adenocarcinoma. L-NAME, an inhibitor of eNOS, inhibits tumorigenic growth in KRas-driven pancreatic cancer [41]. In addition, L-NAME effectively suppresses the metastatic ability of invasive pancreatic cancer cells [46]. Therefore, although we did not perform the experiments in this study, we expect that the combination of a GPR87 inhibitor with an eNOS inhibitor can serve as an attractive therapeutic strategy to achieve clinical benefits in lung adenocarcinoma.

## 5. Conclusions

**Figure 6:**
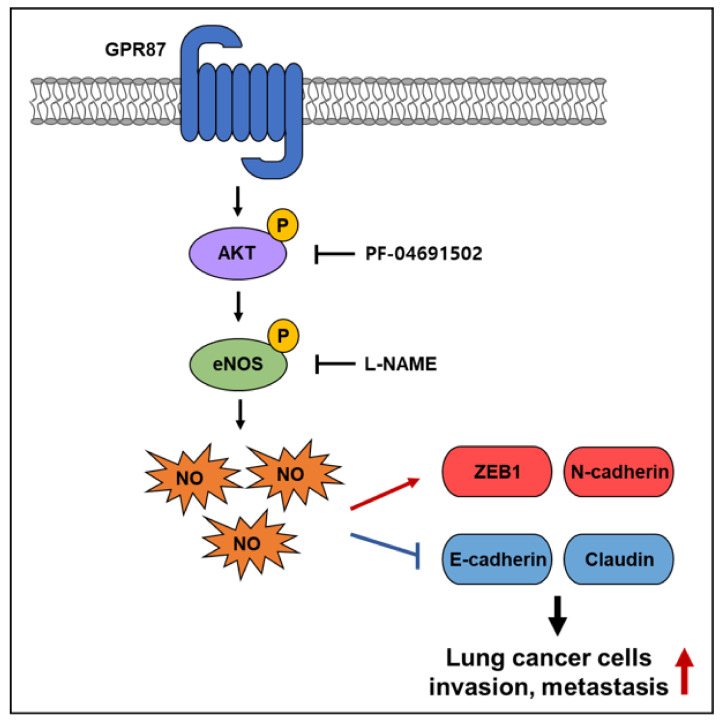
Schematic illustration of the mechanism of GPR87-promoted metastasis in lung adenocarcinoma.

Herein, we demonstrate that GPR87 is upregulated in lung adenocarcinoma and that overexpressed GPR87 contributes to a poor prognosis in lung adenocarcinoma patients. Furthermore, we show that upregulation of GPR87 in lung adenocarcinoma promoted metastatic properties both in vitro and in vivo through the activation of the AKT-eNOS-NO signaling pathway (Figure 6). Our study revealed that GPR87 has oncogenic functions in lung adenocarcinoma and elucidated the novel regulatory mechanisms that promote lung adenocarcinoma progression. Finally, we found that eNOS is an important mediator in the downstream mechanisms regulated by GPR87 in lung adenocarcinoma and, indeed, eNOS plays an oncogenic functional role in cancer cells. These results indicate that the AKT-eNOS-NO pathway may be worth targeting for the treatment of GPR87-overexpressed lung adenocarcinoma.

## Data Availability

All data generated and analyzed during the current study are available from the corresponding author on reasonable request.

## Supplementary Materials

The following are available online at https://www.mdpi.com/article/10.3390/cancers14010019/s1, Figure S1: Uncropped western blot images of Figure 1, Figure S2: Uncropped western blot images of Figure 2, Figure S3: Uncropped western blot images of Figure 3, Figure S4: Uncropped western blot images of Figure 4.
