# Supplementary material for: GPR87 Promotes Metastasis through the AKT-eNOS-NO Axis in Lung Adenocarcinoma
Source: Cancers (Basel). 2021 Dec 21;14(1):19. doi: 10.3390/cancers14010019 (PMC8750422; doi:10.3390/cancers14010019)
Supplement: Supplementary file 1 [file cancers-14-00019-s001.zip › cancers-1490611-supplementary.pdf]

Figure S1 (a)

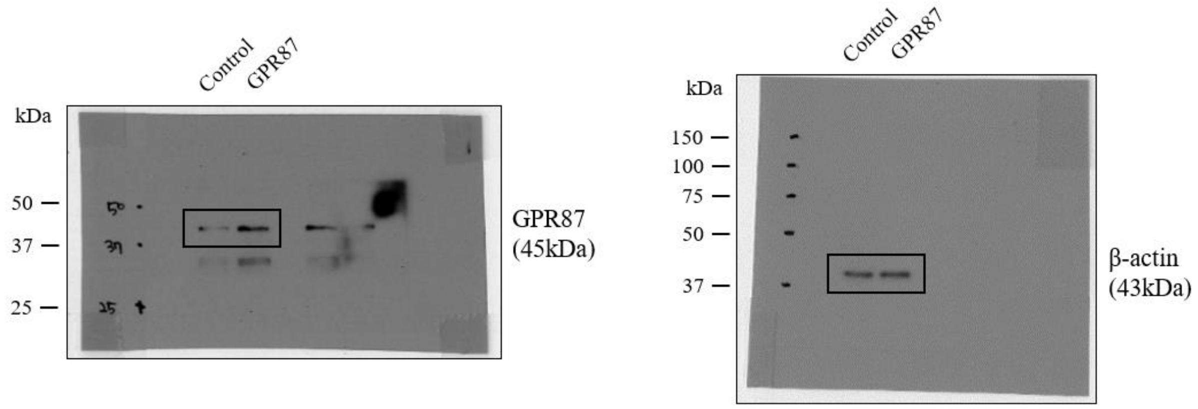

Figure S1 (b)

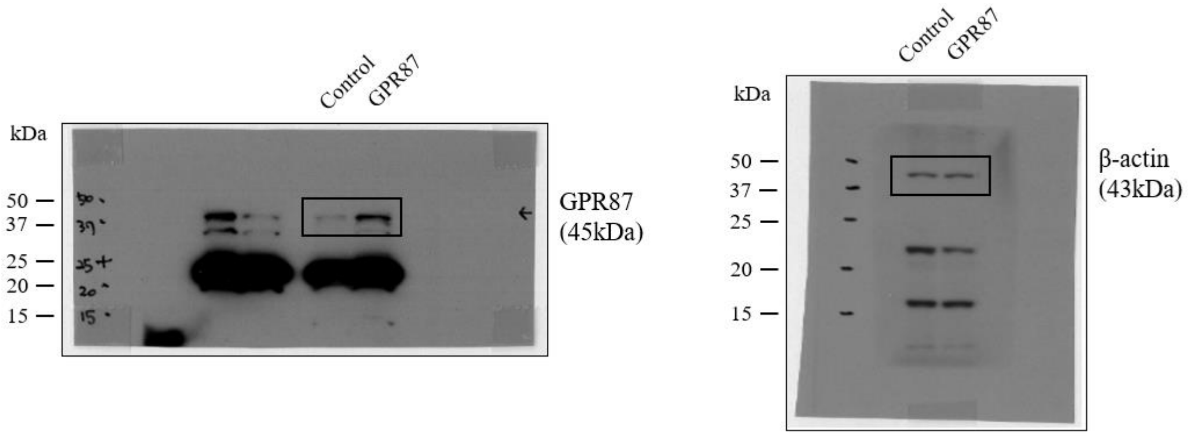

Figure S1 (c)

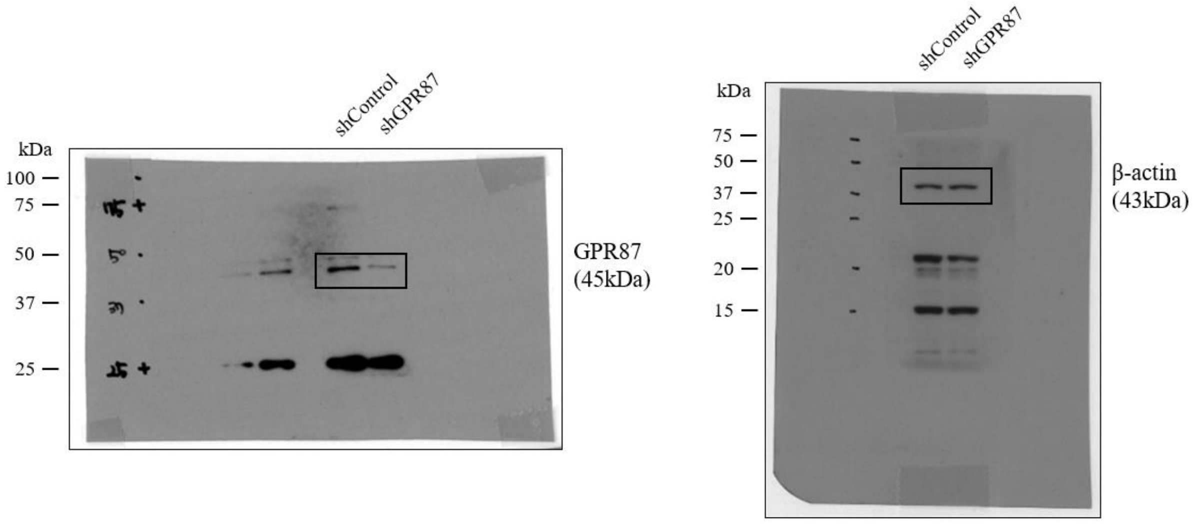

Figure S1 (d)

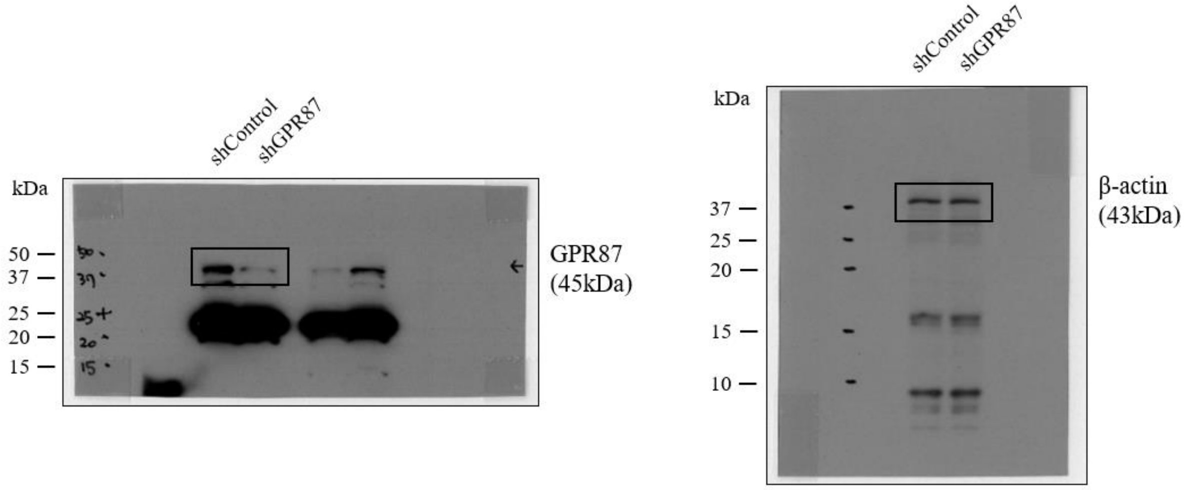

Figure S2 (a)

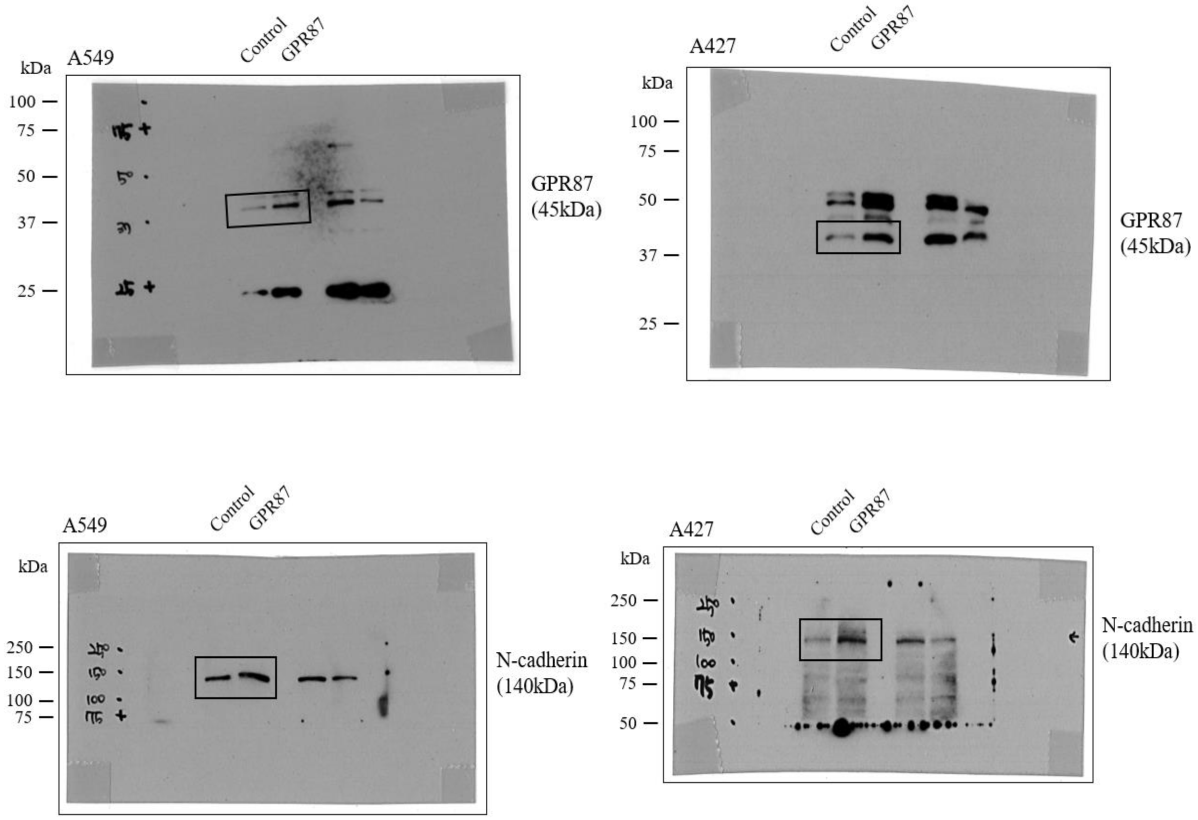

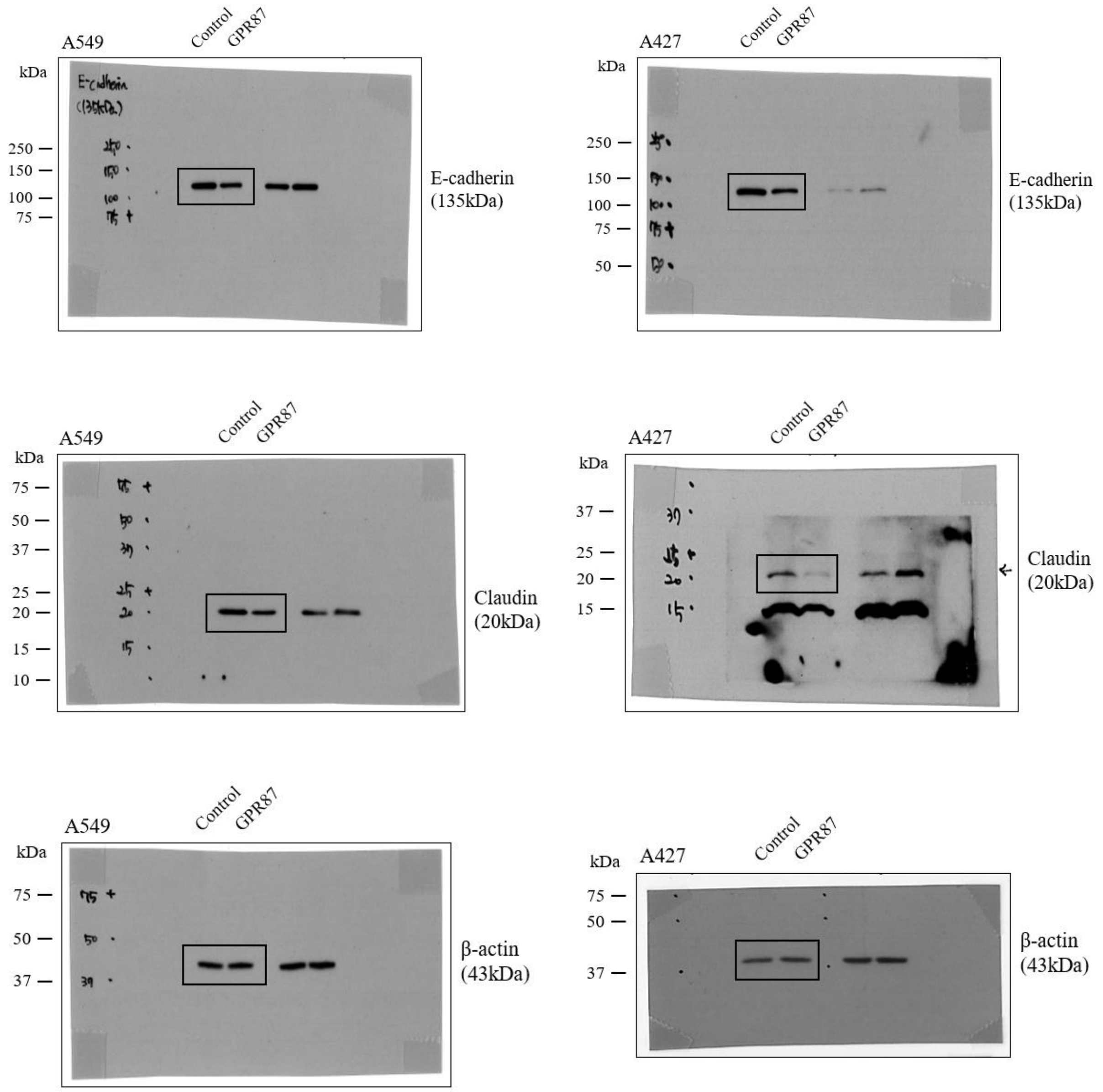
A549
Control
GPR87
kDa
250
150
100
75
E-cadherin
(135kDa)
A427
Control
GPR87
kDa
250
150
100
75
50
E-cadherin
(135kDa)
A549
Control
GPR87
kDa
75
50
37
25
20
15
10
Claudin
(20kDa)
A427
Control
GPR87
kDa
37
25
20
15
Claudin
(20kDa)
A549
Control
GPR87
kDa
75
50
37
β-actin
(43kDa)
A427
Control
GPR87
kDa
75
50
37
β-actin
(43kDa)

Figure S2 (b)

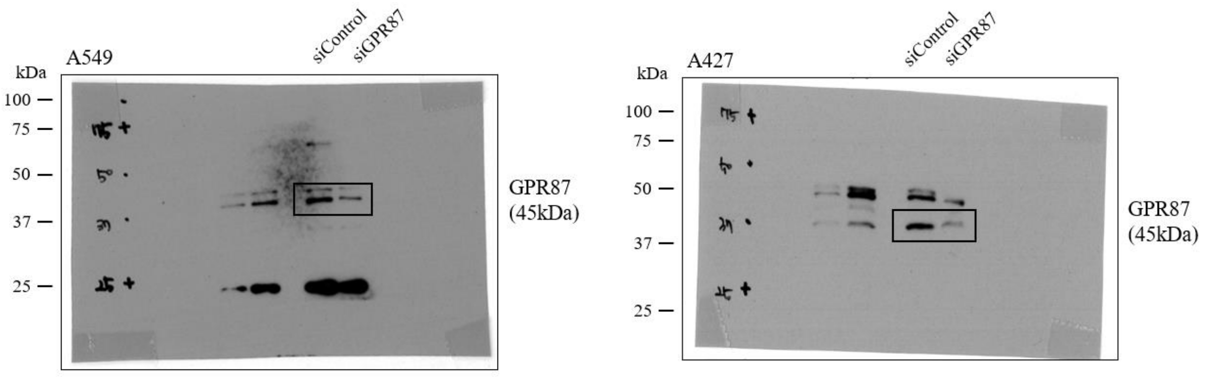
A549
siControl
siGPR87
kDa
100
75
50
37
25
GPR87
(45kDa)
A427
siControl
siGPR87
kDa
100
75
50
37
25
GPR87
(45kDa)

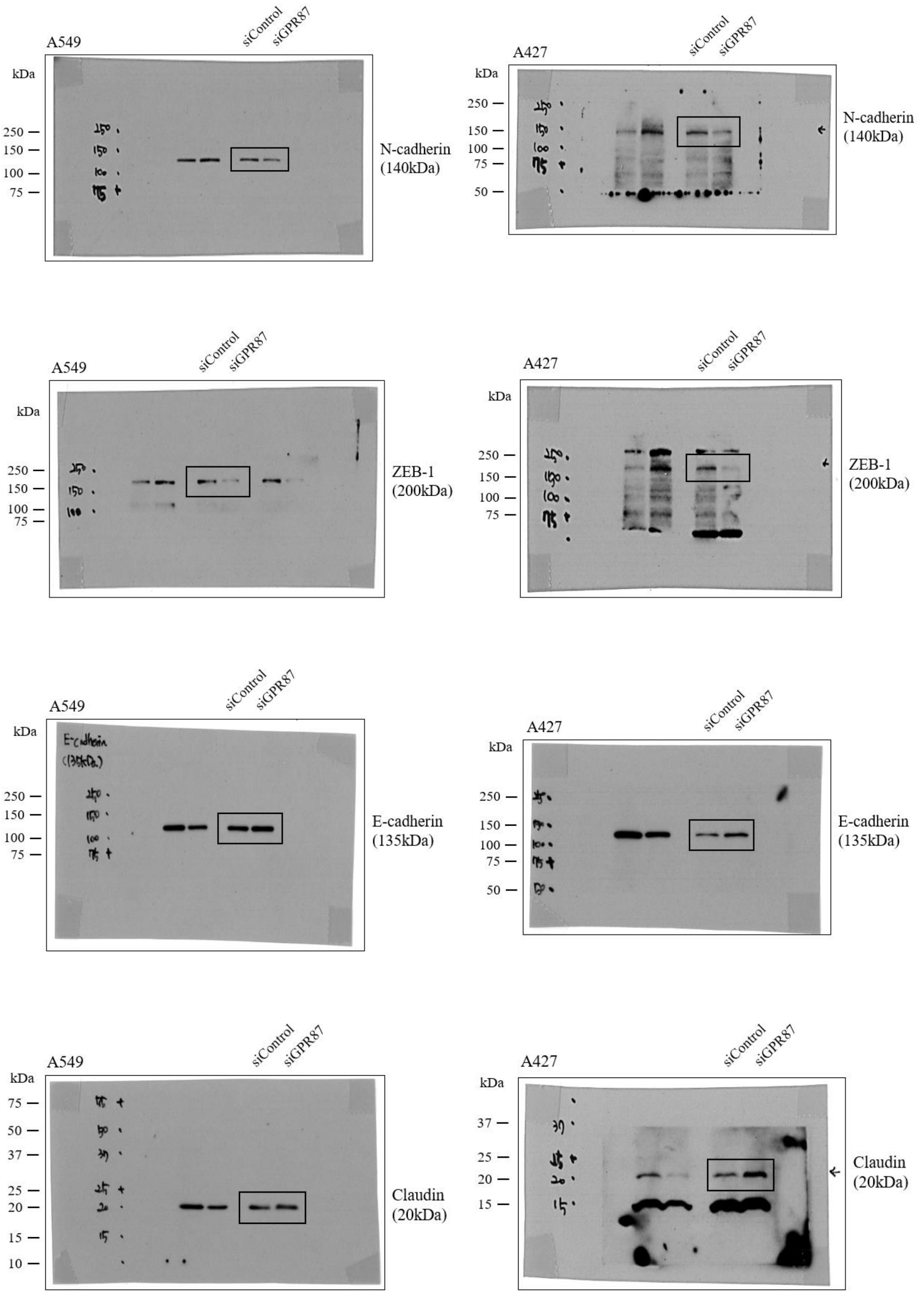
A549
siControl
siGPR87
kDa
250
150
100
75
N-cadherin
(140kDa)
A427
siControl
siGPR87
kDa
250
150
100
75
50
N-cadherin
(140kDa)
A549
siControl
siGPR87
kDa
250
150
100
75
ZEB-1
(200kDa)
A427
siControl
siGPR87
kDa
250
150
100
75
ZEB-1
(200kDa)
A549
siControl
siGPR87
kDa
250
150
100
75
E-cadherin
(135kDa)
A427
siControl
siGPR87
kDa
250
150
100
75
50
E-cadherin
(135kDa)
A549
siControl
siGPR87
kDa
75
50
37
25
20
15
10
Claudin
(20kDa)
A427
siControl
siGPR87
kDa
37
25
20
15
Claudin
(20kDa)

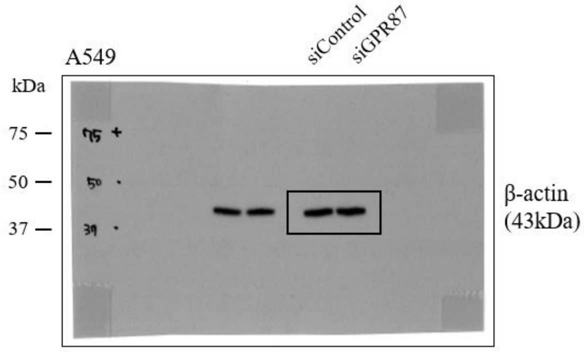

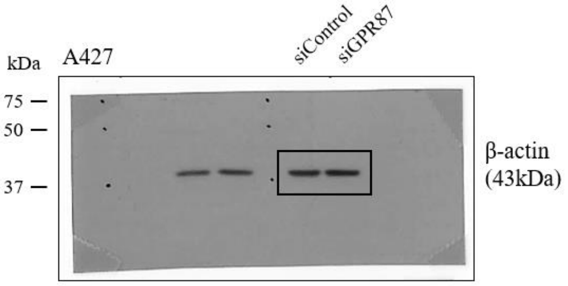

Figure S3 (a) Human phospho-kinase array

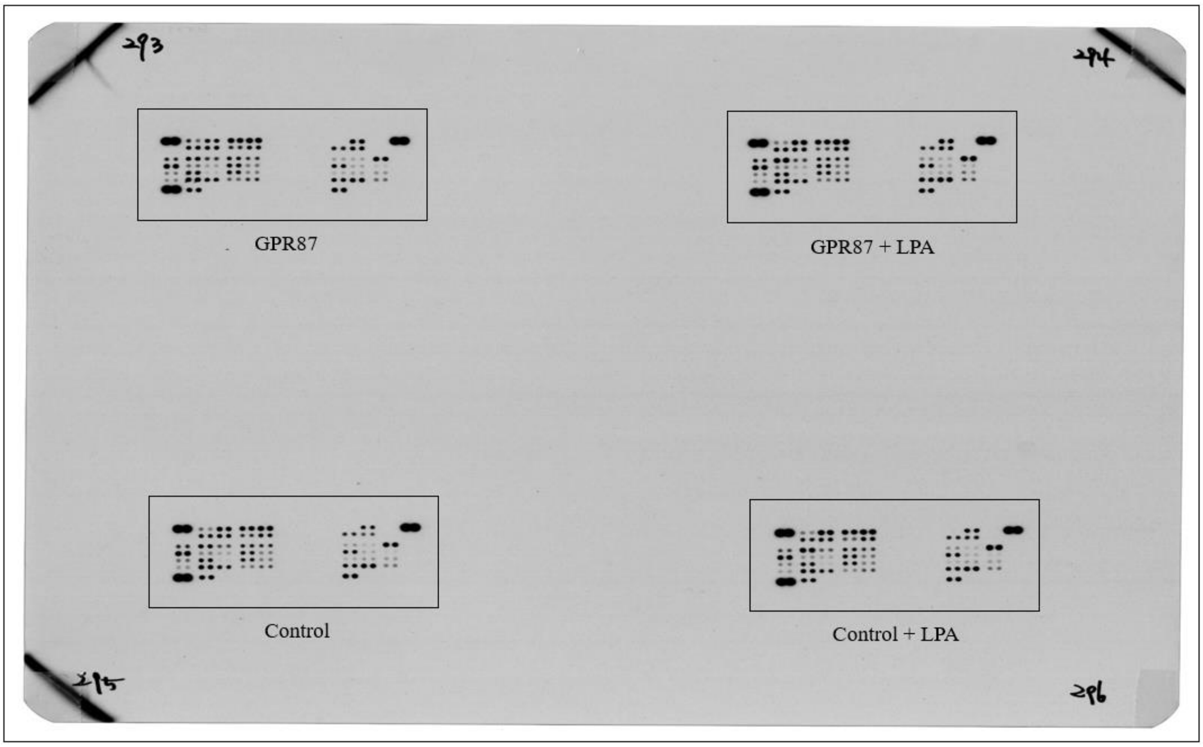

Figure S3 (b)

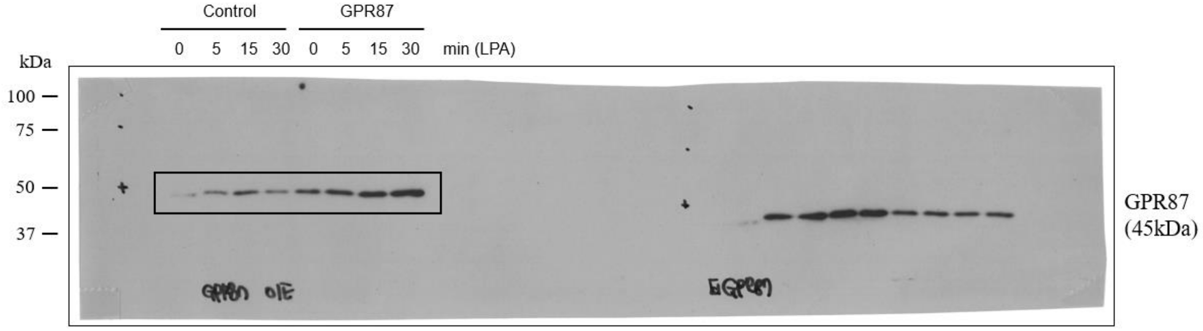

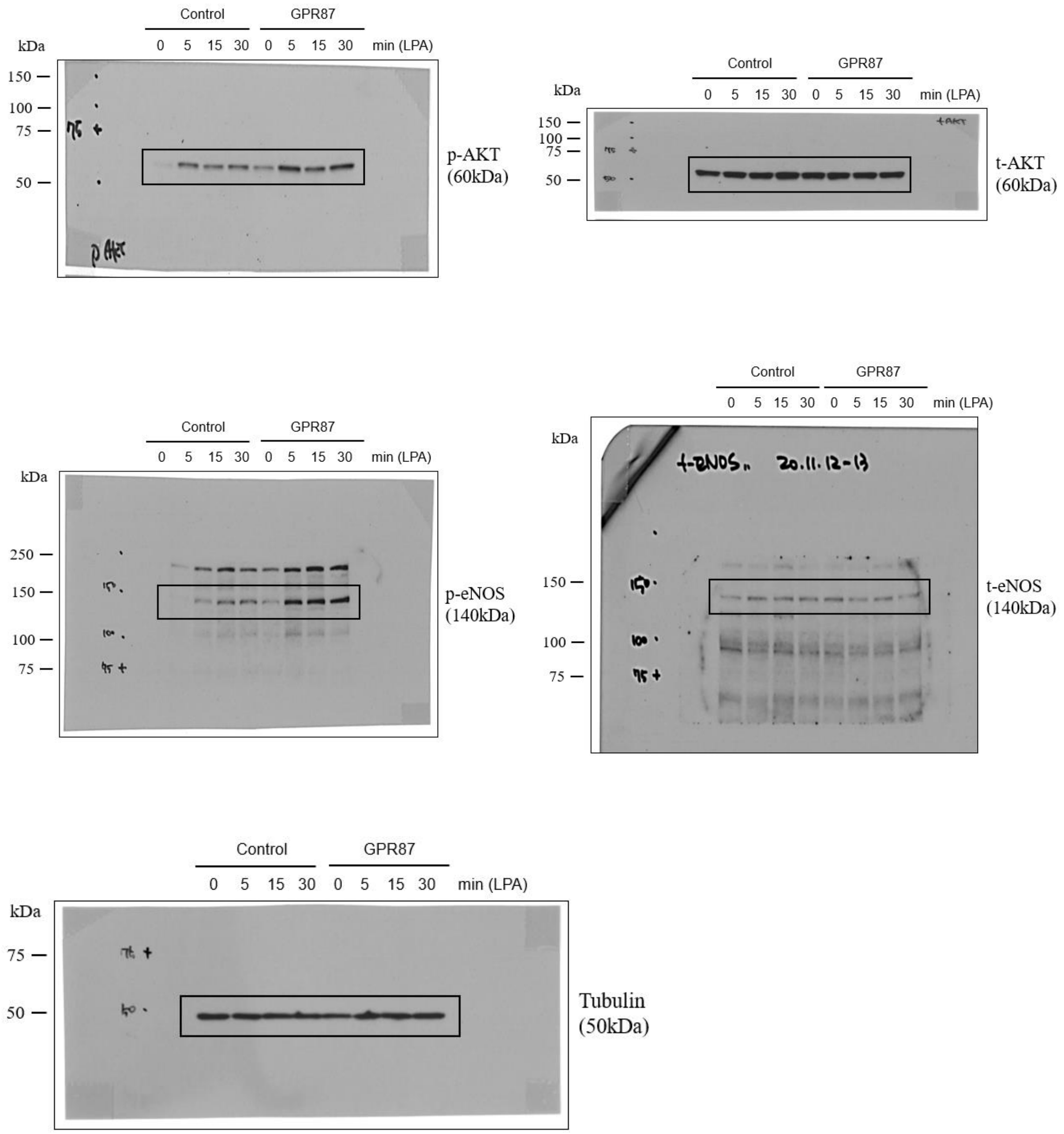
Control
GPR87
kDa
0 5 15 30 0 5 15 30 min (LPA)
150
100
75
50
p-AKT
(60kDa)
t-AKT
(60kDa)
250
p-eNOS
(140kDa)
t-eNOS
(140kDa)
Tubulin
(50kDa)

Figure S3 (c)

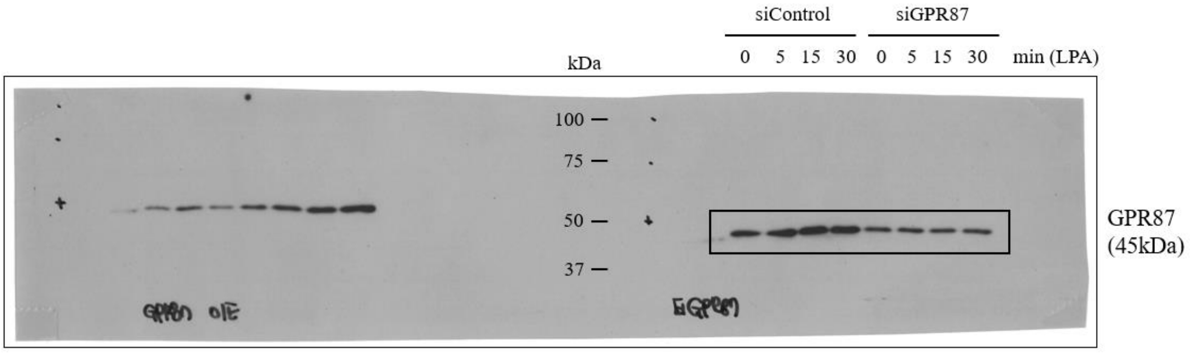
siControl
siGPR87
kDa
0 5 15 30 0 5 15 30 min (LPA)
100
75
50
37
GPR87
(45kDa)

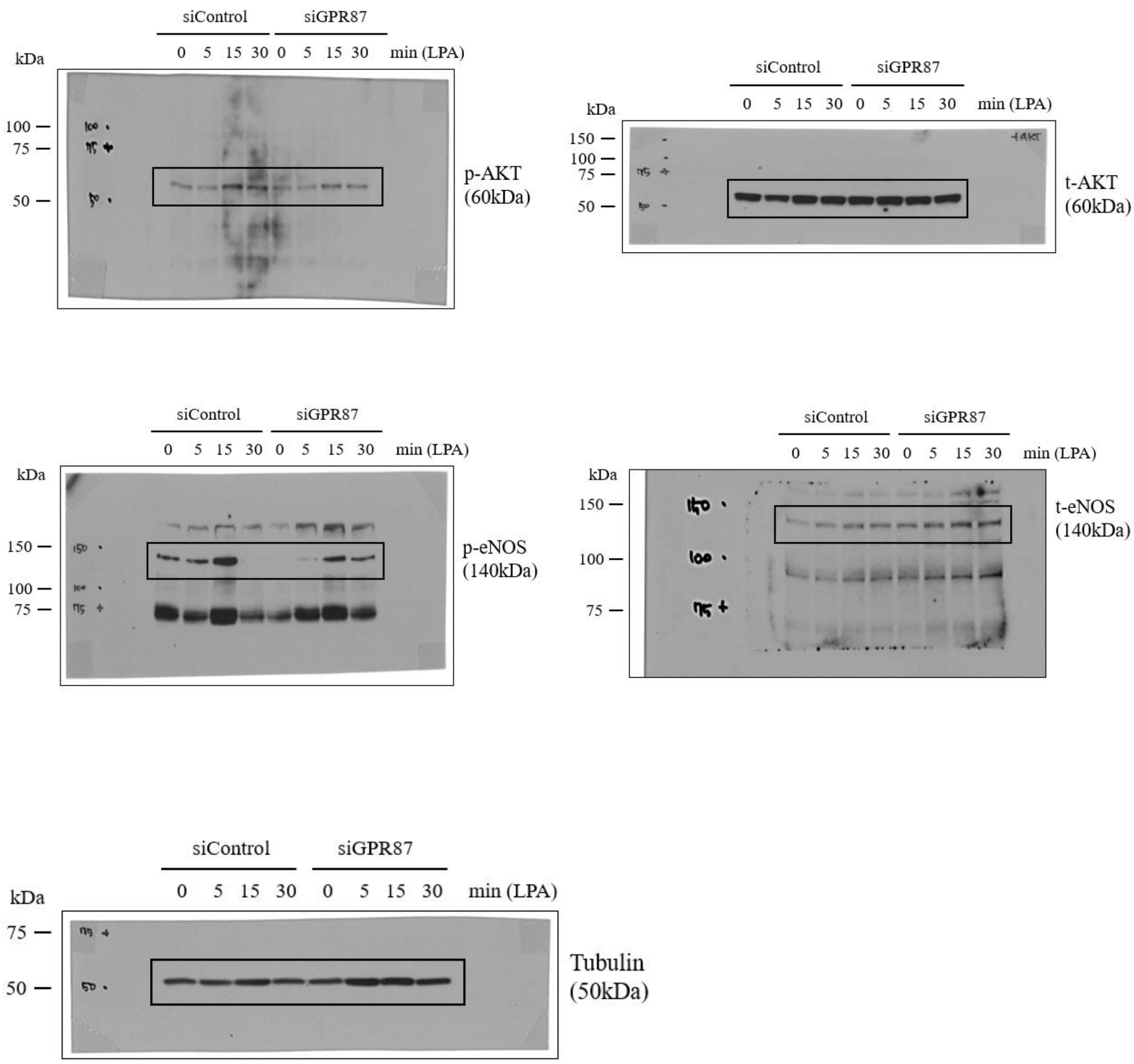
siControl
siGPR87
0 5 15 30 0 5 15 30 min (LPA)
kDa
100
75
50
p-AKT (60kDa)
kDa
150
100
75
50
t-AKT (60kDa)
kDa
150
100
75
p-eNOS (140kDa)
kDa
150
100
75
t-eNOS (140kDa)
kDa
75
50
Tubulin (50kDa)

Figure S4 (c)

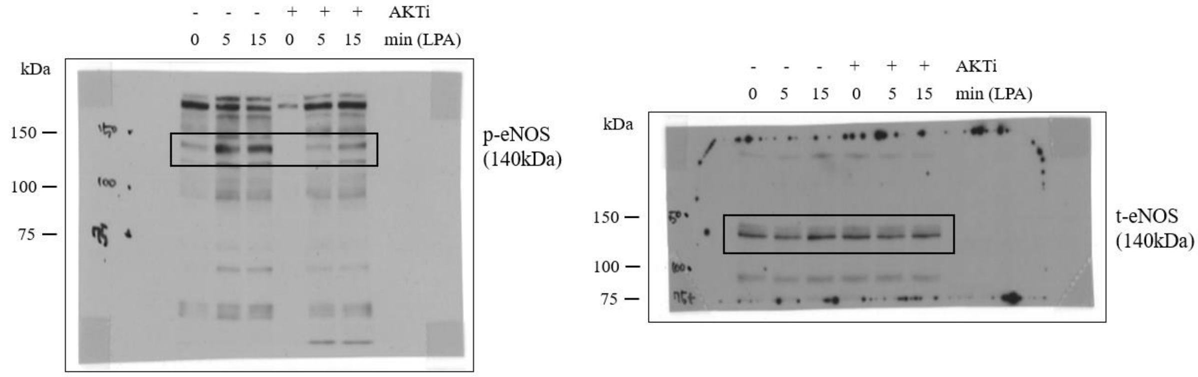
- - - + + + AKTi
0 5 15 0 5 15 min (LPA)
kDa
150
100
75
p-eNOS (140kDa)
kDa
150
100
75
t-eNOS (140kDa)

Figure S4 (g)

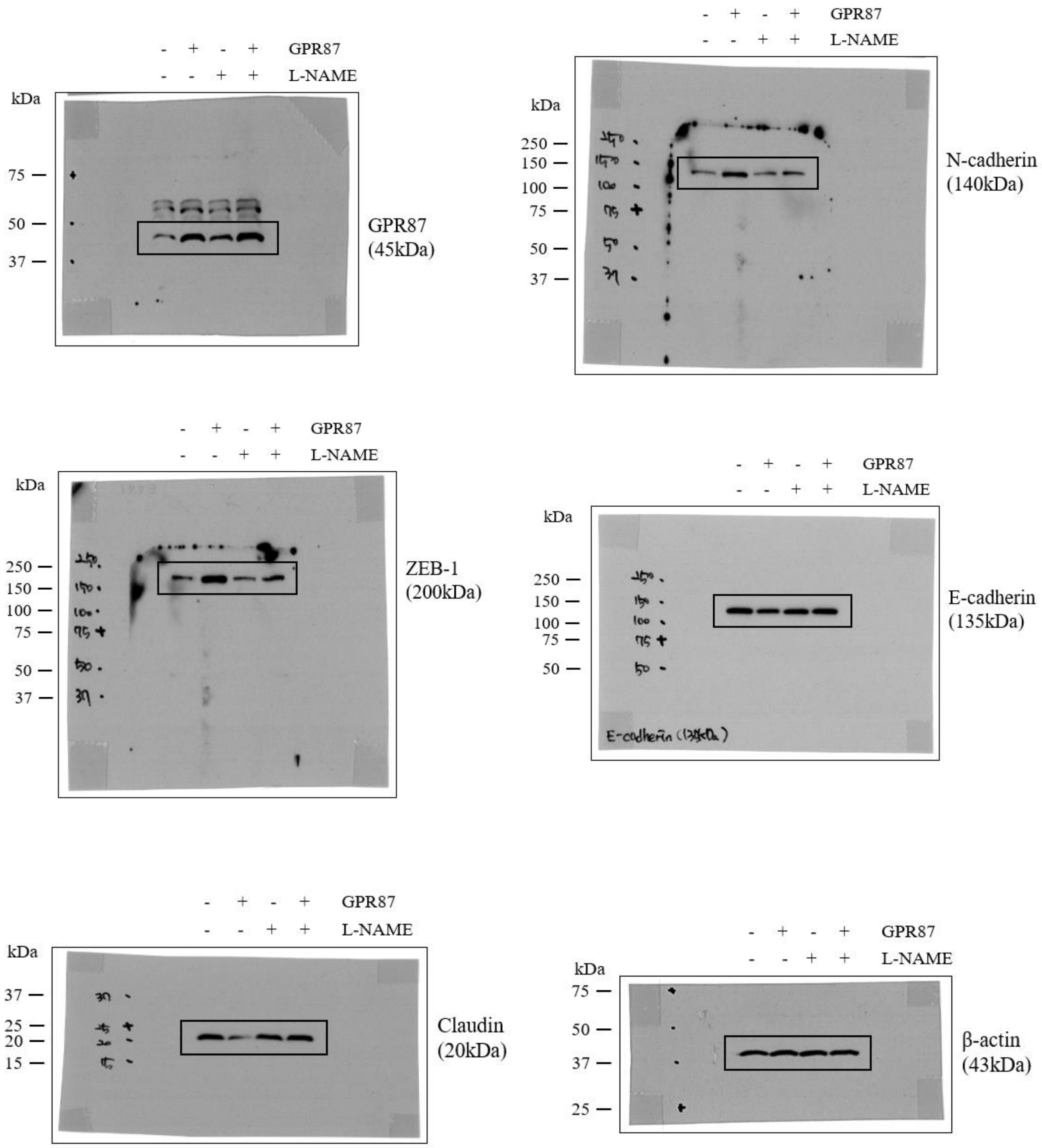

- + - + GPR87
- - + + L-NAME
kDa
75
50
37
GPR87
(45kDa)
- + - + GPR87
- - + + L-NAME
kDa
250
150
100
75
50
37
N-cadherin
(140kDa)
- + - + GPR87
- - + + L-NAME
kDa
250
150
100
75
50
37
ZEB-1
(200kDa)
- + - + GPR87
- - + + L-NAME
kDa
250
150
100
75
50
E-cadherin
(135kDa)
- + - + GPR87
- - + + L-NAME
kDa
37
25
20
15
Claudin
(20kDa)
- + - + GPR87
- - + + L-NAME
kDa
75
50
37
25
β-actin
(43kDa)
